# A Case of Focal Dermal Hypoplasia (Goltze Syndrome) Masquerading as Lingual Tonsillar Hypertrophy

**DOI:** 10.1155/2019/9536256

**Published:** 2019-06-18

**Authors:** Samuel T. Roberts, Greg Shein, Ian Jacobson

**Affiliations:** ^1^Sydney Children's Hospital, Randwick, NSW 2031, Australia; ^2^The University of Newcastle, Callaghan, NSW 2308, Australia

## Abstract

Focal dermal hypoplasia is a rare condition affecting organ systems of mesodermal origin. We present a rare case of this condition presenting with apparent palatine tonsillar regrowth and outline our management. Airway management should always be a consideration in this rare condition.

## 1. Introduction

Focal dermal hypoplasia (Goltze syndrome) is a rare, X-linked autosomal dominant systemic condition which causes dysfunction of multiple organ systems of mesoectodermal origin [1–3]. We present a case of this syndrome masquerading as lingual tonsillar hypertrophy in a 7-year-old female.

## 2. Case Presentation

**Figure 1:**
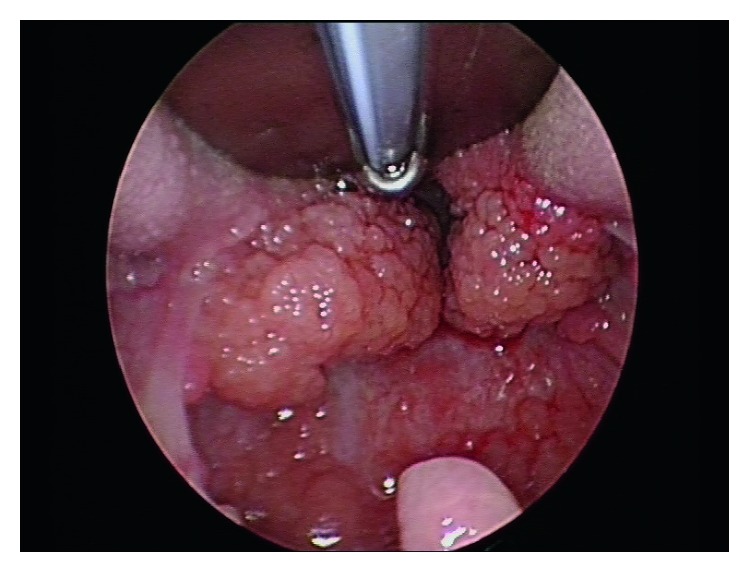
Intraoperative photograph demonstrating significant enlargement over the area of the lingual tonsil prior to surgical reduction.

**Figure 2:**
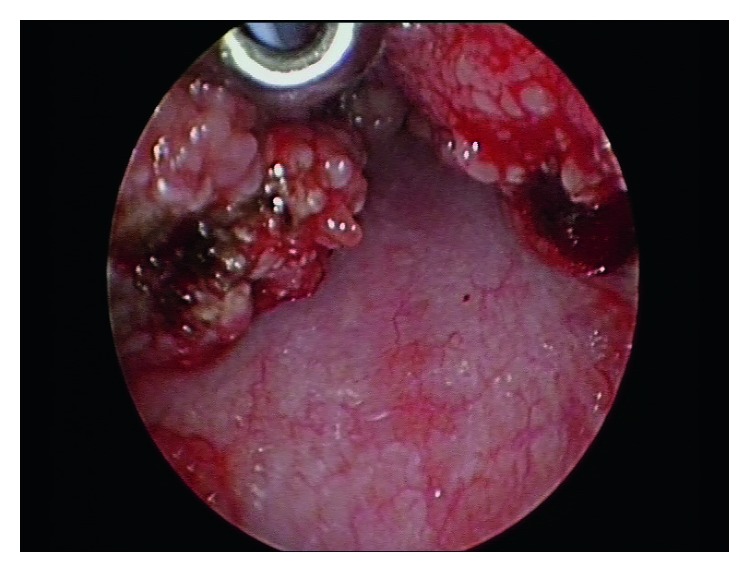
Intraoperative photograph demonstrating the area after surgical removal of the obstructive lesions.

**Figure 3:**
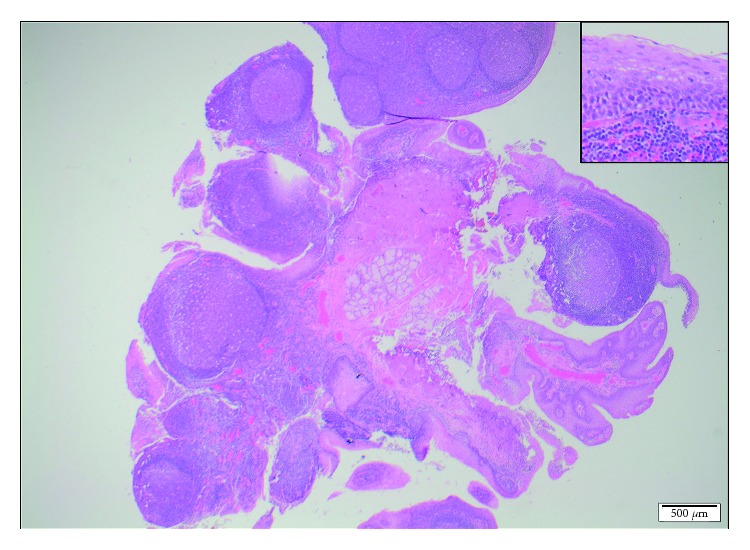
Histology of polyps from base of tongue. The lamina propria contains prominent reactive lymphoid follicles with large germinal centres (original magnification ×20). The overlying squamous epithelium shows no atypia (inset).

**Figure 4:**
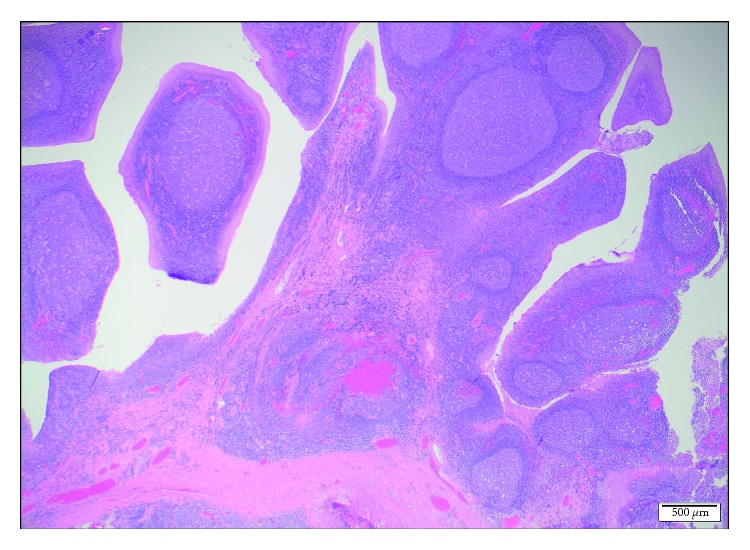
Histology of polyps from base of tongue. The lamina propria contains prominent reactive lymphoid follicles with large germinal centres. The overlying squamous epithelium shows no atypia (original magnification ×20).

A female patient with a diagnosis of Goltze syndrome underwent an adenotonsillectomy at the age of two for obstructive sleep apnoea. Prior to this admission, documented manifestations included mild skin changes only. At the time of the adenotonsillectomy procedure, note was made of papillomas in the oropharynx and large “granular, papillomatous” tonsils. This procedure was a monopolar comprehensive extracapsular tonsillectomy with curette adenoidectomy. The papilloma histology revealed Schneiderian-type papilloma, and the tonsillar pathology revealed reactive lymphoid hyperplasia. At age 4, she underwent a laryngobronchoesophagoscopy after returning to the clinic with worsening oropharyngeal papillomata and excessive throat clearing. This procedure revealed papilloma growth in the oropharynx, large lingual tonsils with tongue base papillomas, but no papillomata were identified on the vocal cords. Oesophagoscopy also revealed papillomata throughout the oesophagus. Two years later, she presented to an emergency department for an unrelated matter and was thought to have had palatine tonsillar regrowth. She was referred back the ENT service, and another laryngobronchoeosophagoscopy was arranged to further evaluate the nature of the new lesions. This procedure revealed very large papillomatous disease arising from the tongue base and lateral pharyngeal wall with a normal larynx apart from cobblestoning of the larynx and bronchi (Figure 1). The tongue base lesions were resected using monopolar diathermy with relief of airway embarrassment (Figure 2). The specimen was sent for histopathology and virotyping, which revealed reactive lymphoid hyperplasia with mildly thickened and parakeratotic squamous epithelium (Figures 3 and 4). Virology for HPV was negative. She recovered well and was discharged the following day. At follow-up, she was doing well and was referred to the infectious disease team for consideration of vaccination or antiviral treatment. She will be followed-up regularly, given the risk of recurrence.

## 3. Discussion

Goltze syndrome is a rare X-linked genetic condition caused by a mutation of the PORCN gene, of which 80 mutations have been recorded [3, 4]. Its presentation can affect areas of mesodermal and ectodermal origin in multiple body systems and holds significant variation between individuals, though skin involvement is regarded as essential [1, 5, 6]. Skin manifestations include areas of hypoplastic skin with abnormal red or pink pigmentation and papillomatous growths [3, 5]. Skeletal manifestations include syndactyly, oligodactyly, polydactyly, split-hand/foot, camptodactyly, and reduction of long bones [4]. Other manifestations can include ocular signs (microphthalmia or anophthalmia, colobomas, cataracts, and lacrimal duct abnormalities), dental changes, cleft lip/palate, short stature, and mental retardation [3, 4, 7]. Oropharyngeal manifestations are less common, but can include papilloma on the tongue, tonsils, soft palate, hypopharynx, and larynx [3, 7]. Other case reports have also made note of squamos papilloma of the oesophagus [8]. There are 5 reports in the literature of oropharyngeal papillomatous lymphoid hyperplasia, and 4 of these occurred in young females as with our patient. As in our patient, the vocal folds themselves were free of papilloma. Luckily, the potential threat of airway embarrassment from supraglottic obstruction had been anticipated and prepared for in the management of our patient during both surgeries. Like other case reports, our patients virotyping was negative for HPV [9]. Other case reports have highlighted the importance of anticipating airway embarrassment in the management of patients with this rare condition [3, 10]. With the potentially devastating consequences of airway loss, we support the recommendation of other authors that ENT involvement either preoperatively or during induction of anaesthesia is mandatory.
